# The clinical efficacy of biportal endoscopy is comparable to that of uniportal endoscopy *via* the interlaminar approach for the treatment of L5/S1 lumbar disc herniation

**DOI:** 10.3389/fsurg.2022.1014033

**Published:** 2022-09-27

**Authors:** Rujun Zuo, Yi Jiang, Ming Ma, Shuai Yuan, Jian Li, Chang Liu, Jiexun Zhang

**Affiliations:** Department of Orthopedics (Minimally Invasive Spine Surgery Branch), Beijing Haidian Hospital (Haidian Section of Peking University Third Hospital), Beijing, China

**Keywords:** spinal endoscopy, minimally invasive, biportal endoscopic spine surgery, lumbar disc herniation, operative time, operative blood loss

## Abstract

**Objective:**

To compare the clinical outcomes of unilateral biportal endoscopy/biportal endoscopic spinal surgery (UBE/BESS) *via* the posterior approach with those of interlaminar endoscopic lumbar discectomy (IELD) for the treatment of L5/S1 lumbar disc herniation.

**Methods:**

We collected the clinical data of patients with L5/S1 lumbar disc herniation who had undergone endoscopic surgery at our center from January 2020 to July 2021, and 92 patients were included. They were divided into UBE/BESS (*n* = 42) and IELD (*n* = 50) groups. The incision length, operative time (overall operative, extracanal operative, and intracanal decompression times), intraoperative radiation exposure dose, changes in hemoglobin before and after surgery, postoperative hospital stay, visual analog scale (VAS) score for low back pain and leg, and Oswestry disability index (ODI) were statistically analyzed.

**Results:**

One case incurred dural tear in the UBE/BESS group, and one case developed recurrence in the IELD group. Postoperatively, the VAS score and ODI index decreased significantly in both groups (*P* < 0.01). VAS and ODI scores (preoperative as well as 3 days, 3 months, 6 months, and 12 months after surgery), the overall operative time, and postoperative hospital stay were not significantly different between the two groups (*P* > 0.05). No statistical difference in intraoperative radiation exposure dose was noted between the two groups (*P* > 0.05). The surgical incision length was greater in the UBE/BESS group (*P* < 0.01), and pre- and postoperative hemoglobin changes were more pronounced in the UBE/BESS group (*P* < 0.01). The UBE/BESS group had a longer extracanal operative time and shorter intracanal decompression time (*P* < 0.01).

**Conclusions:**

The clinical efficacy of UBE/BESS for L5/S1 lumbar disc herniation is comparable to that of IELD. Intraoperative radiation exposure doses were similar in both techniques. UBE/BESS required more time to identify tissue structures and a larger working space when operating outside the spinal canal; however, the efficiency of nucleus pulposus removal and nerve root release inside the spinal canal superseded that in IELD. Furthermore, the surgical incision in the UBE/BESS technique was longer, with greater actual blood loss during surgery, thus rendering UBE/BESS inferior to the IELD technique in terms of surgical trauma. Nonetheless, no significant difference was noted between the two techniques in the postoperative recovery time of patients.

## Introduction

Lumbar disc herniation is a disease that is commonly encountered in spine surgery, and it is the main cause of low back pain and lower-limb radiating pain, with a prevalence of 11%–13% in China (1, 2). Although most lumbar disc herniations can be relieved using conservative treatment (3, 4), 25% of affected patients still require surgery for recurrent symptoms (5). With the continuous advancement of medical technology, minimally invasive surgical techniques have gradually become an important part of the stepwise treatment process for lumbar disc herniation (6). In recent years, percutaneous uniportal endoscopic surgery has been widely used, achieving favorable clinical results (7–11). Interlaminar endoscopic lumbar discectomy (IELD) has exhibited immense technical advantages for L5/S1 lumbar disc herniation (12–17). However, the equipment **involved** is expensive and difficult to master (18, 19). In recent years, unilateral biportal endoscopy/biportal endoscopic spinal surgery (UBE/BESS) has emerged, providing a novel option for the minimally invasive endoscopic treatment of lumbar disc herniation (20, 21). Its use has rapidly proliferated owing to widely available equipment and the vast similarity of its surgical concept to that of conventional surgery. Few studies have compared the two techniques for the treatment of lumbar disc herniation. Therefore, to explore the differences between the two surgical techniques, this study examined and compared the clinical efficacy of UBE/BESS with that of IELD in the treatment of L5/S1 lumbar disc herniation *via* the interlaminar approach.

## Materials and methods

### Inclusion and exclusion criteria

The inclusion criteria were as follows: (1) a clear diagnosis of L5/S1 lumbar disc herniation with significant lower extremity radiating pain, low back pain, and lower extremity motor and/or sensory dysfunction; (2) computed tomography scan and magnetic resonance image (MRI) of the lumbar spine consistent with clinical symptoms and signs; and (3) treatment with systematic conservative treatment for a duration >3 months. The exclusion criteria were as follows: (1) history of lumbar spine surgery at the L5/S1 segment; (2) lumbar spine infection, tumor, or trauma; (3) presence of lumbar instability and/or lumbar isthmic fracture; (4) concomitant severe psychiatric disorders; and (5) inability to tolerate general anesthesia.

### Patients

This was a retrospective cohort study wherein the data of 92 patients with L5/S1 lumbar disc herniation treated at our center from January 2020 to July 2021 using spinal endoscopic surgery *via* the interlaminar approach were collected. The patients were recruited based on the inclusion and exclusion criteria, and they were all followed up for ≥12 months, mean 13.26 months. The preoperative clinical manifestations were low back pain, lower extremity radiating pain, and lower extremity motor and/or sensory dysfunction, and all patients had preoperative MRIs confirming the diagnosis of lumbar disc herniation (L5/S1).

UBE/BESS group: 23men, 19women, mean age was 45.57 ± 11.15 years (25–66 years), mean body mass index (BMI) was 24.53 ± 2.96. IELD group: 31men, 19women, mean age was 46.68 ± 12.09 years (22–67 years), mean BMI was 24.57 ± 3.71.

The study was conducted in accordance with the principles of the Declaration of Helsinki, and patients or their families provided written informed consent for the procedure.

### Surgical technique

All surgical procedures were performed by the same experienced surgeon. Patients in both groups were placed in the prone position and underwent surgery under general anesthesia, and no postoperative drains were utilized in either group.

#### UBE/BESS

**Figure 1:**
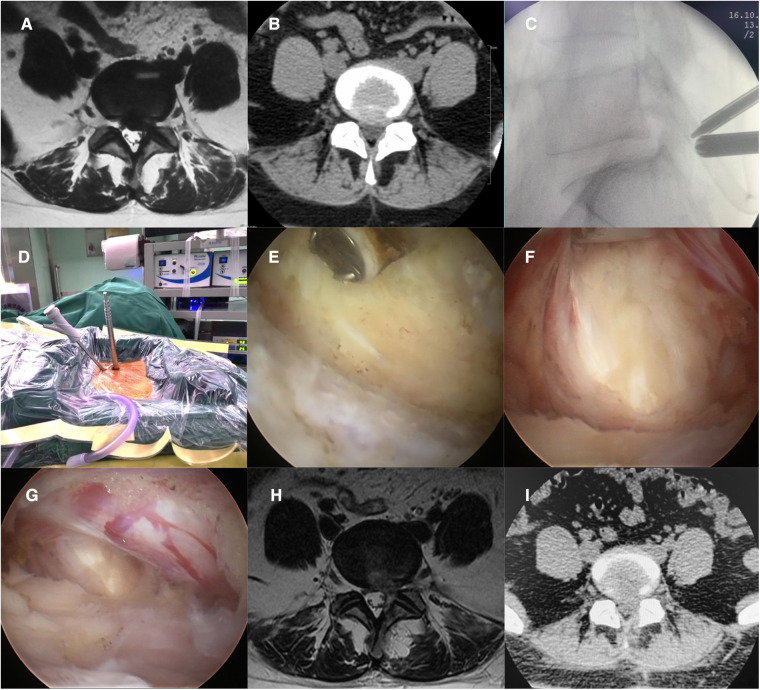
(**A,B**) Preoperative patient MRI and CT revealing L5/S1 disc herniation. (**C,D**) Intraoperative localization of UBE/BESS and establishment of proximal and distal access. (**E**) Establishment of a working space to expose the interlaminar window. (**F**) Medial retraction of the nerve root and exposure of the herniated disc. (**G**) Loosened nerve roots after disc removal. (H, I) Postoperative patient MRI and CT showed that the L5/S1 herniated disc had been removed.

The inferior border of the affected L5 pedicle and superior border of the S1 pedicle were located using a C-arm and marked on the body surface. A longitudinal skin incision of approximately 5 mm was made at the proximal marker point, and an endoscopic puncture sheath was placed. A transverse skin incision of approximately 10 mm was made at the distal marker point, and a progressive dilator was placed. The puncture sheath and the dilator met together at the L5 spinous process and plate migration, and C-arm fluoroscopy was used to confirm the position. The dilator was subsequently removed and a periosteum detacher placed. After placement of the endoscope, the inferior margin of the L5 lamina was identified using radiofrequency hemostasis, followed by exposure of the interlaminar window. If necessary, the lamina was partially shaped using a grinding drill. The ligamentum flavum was incised at the medial edge of the articular eminence under endoscopic surveillance, and the canal was entered. After entering the spinal canal, the lateral margin of the S1 nerve root was revealed and the S1 nerve root can be retracted in the midline using a nerve puller to reveal the herniated disc. The herniated disc was removed, and the procedure was completed with adequate neurological decompression and hemostasis (Figure 1).

#### IELD

**Figure 2:**
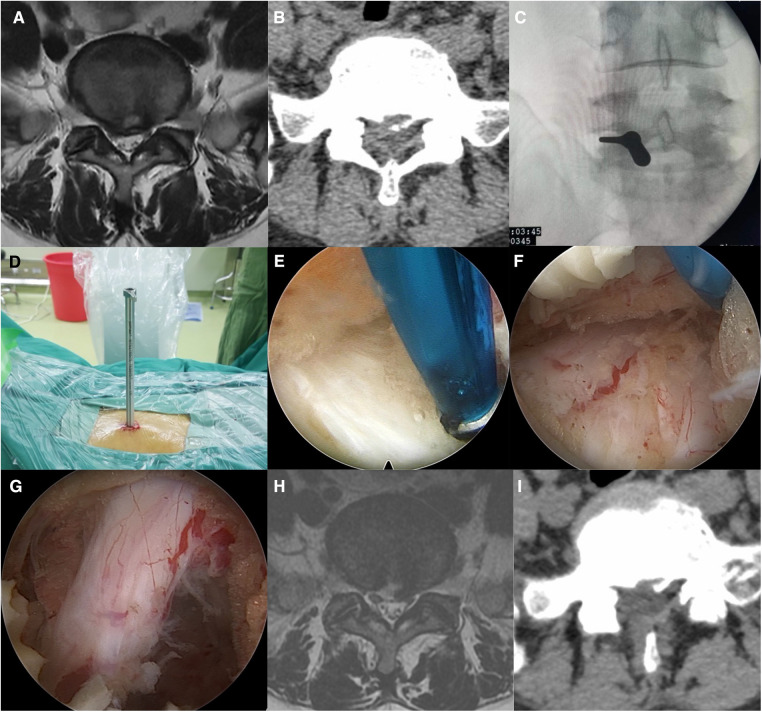
(**A,B**) Preoperative patient MRI and CT revealing L5/S1 disc herniation. (**C,D**) Intraoperative positioning and access establishment. (**E**) Exposure of the interlaminar window. (**F**) Exposure of the prolapsed disc using a working tube in the axilla of the nerve root. (**G**) Loosened nerve roots after disc removal. (**H,I**) Postoperative patient MRI and CT showed that the L5/S1 herniated disc had been removed.

Using a 6.9-mm endoscopic system, the center of the L5/S1 interlaminar window on the affected side was positioned under the C-arm and marked on the body surface. A longitudinal skin incision of approximately 7 mm was made at the marking point, a stepwise dilator and working tube were placed directly on the surface of the L5/S1 interlaminar window, and the position of the working tube was confirmed using C-arm fluoroscopy. After placement of the endoscope, the interlaminar window and ligamentum flavum were exposed. The superficial ligamentum flavum was removed using nucleus pulposus forceps. Subsequently, the deep ligamentum flavum was bluntly separated using a nerve stripper, and the ligamentum flavum was split using a working tube to facilitate entry into the spinal canal. The ligamentum flavum was removed to the medial edge of the facet joint using punches, and the dural sac and nerve root were exposed. Based on the location of the herniated disc, the disk fragment was removed in the axilla of the nerve root or shoulder (Figure 2).

### Postoperative management

Postoperative analgesic treatment was routinely administered. If no dural tear occurred, the patient could walk after 4 h post surgery; however, if a dural tear occurred, no special treatment was administered to asymptomatic patients, and bed rest for 5–7 days was prescribed for symptomatic patients. The patient's lumbar spine MRI was reviewed before discharge from the hospital. The patient was advised to wear a lumbar brace for 1 month and avoid strenuous activities for 3 months after surgery. Routine blood tests were performed 3 days after surgery, and the hemoglobin (Hb) level was recorded.

### Observation indicators

The operative time was recorded for all patients and categorized as follows: (1) overall operative time, (2) extracanal operative time (time from skin incision to entry into the spinal canal), (3) intracanal decompression time (time from entry into the spinal canal to the end of the operation), (4) intraoperative radiation exposure dose, (5) operative incision length (measured as the sum of the two proximal and distal incision lengths in the UBE/BESS group), (6) operative related complications, (7) postoperative hospital stay, and (8) preoperative and postoperative day 3 Hb levels. The visual analog scale (VAS) scores of back/leg pain before and 3 days, 3 months, 6 months, and 12 months after surgery as well as the Oswestry disability index (ODI) before and 3 months, 6 months, and 12 months after surgery were recorded.

### Statistical analysis

SPSS (version 26.0; IBM SPSS Inc., Chicago, IL, USA) software was used for statistical analyses. Normally distributed measures are expressed as the mean ± standard deviation ( ± s). Patient age, operative time, incision length, Hb, and postoperative hospital stay were compared between groups using the independent-samples *t-test* or independent-samples nonparametric test. Hb levels, as well as VAS and ODI scores at different time points, were compared within groups using the paired-samples *t-test* or paired-samples nonparametric test. The *χ*^2^ test was used to compare results between sexes among the patients in the two groups. Statistical significance was set at *P* < 0.05.

## Results

### Baseline information

Baseline information, such as age, sex, preoperative low back/leg VAS score, and ODI were not statistically significantly different between the two groups (*P* > 0.05), as shown in Table 1.

**Table 1:** Baseline information of UBE/BESS and IELD.

Group	UBE/BESS (*n* = 42)	IELD (*n* = 50)	Statistical values	*P*
Age (years)	45.57 ± 11.15	46.68 ± 12.09	*t *= −0.454	>0.05
Male	23	31	*χ*^2^ = 0.493	>0.05
Female	19	19		
Preoperative low back pain VAS	3.95 ± 3.00	3.22 ± 2.88	Z = −1.204	>0.05
Preoperative leg pain VAS	8.14 ± 1.26	7.82 ± 1.7	Z = −0.497	>0.05
Preoperative ODI (%)	66.07 ± 13.48	71.48 ± 15.94	t = −1.74	>0.05

### Perioperative outcomes of UBE/BESS and IELD

Patients in both groups underwent surgery successfully. On comparing the two groups, the surgical incision length in the UBE/BESS group was significantly longer than that in the IELD group (*P* < 0.01). A significant difference in Hb level before and after surgery was noted in the UBE/BESS group compared with that in the IELD group (*P* < 0.01), suggesting that the actual blood loss in the UBE/BESS group was greater than that in the IELD group. No statistical difference in the total operative time was observed between the two groups (*P* > 0.05); however, the extracanal operative time was significantly longer in the UBE/BESS group than in the IELD group (*P < 0*.01), and the operative time for intracanal decompression was significantly shorter in the UBE/BESS group than in the IELD group (*P < 0*.01). The Hb level on postoperative day 3 was significantly different from the preoperative Hb level (*P* < 0.01) in both groups, as shown in Table 2.

**Table 2:** Comparison of perioperative date of UBE/BESS and IELD.

	UBE/BESS	IELD	Statistical values	*P*
Total operation time (min)	68.57 ± 10.87	65.6 ± 15.24	t = 1.057	>0.05
Extracorporeal operation time (min)	31.12 ± 4.48	15.84 ± 2.88	t = 19.028	<0.01
Intradural decompression time (min)	37.45 ± 12.32	49.76 ± 14.73	t = −4.295	<0.01
Length of surgical incision (mm)	14.93 ± 1.30	7.46 ± 1.11	Z = −8.293	<0.01
Intraoperative radiation exposure dose (mGy)	0.72 ± 0.11	0.77 ± 0.14	Z = −1.508	>0.05
Preoperative Hb (g/L)	144.79 ± 13.76	138.48 ± 14.33	Z = −1.435	>0.05
Hb (g/L) on the third postoperative day	134.52 ± 13.45*	136.60 ± 14.17**	t = −0.716	>0.05
Hb change (g/L)	10.26 ± 3.21	1.88 ± 1.573	Z = −8.045	< 0.01
Post-operative hospital stay (days)	6.88 ± 1.85	7.36 ± 4.62	Z = −0.812	>0.05

Note: In the intra-group comparison of the two groups, the difference in Hb on the third postoperative day in the UBE/BESS group compared with the preoperative Hb was significant,*P < 0.01; the difference in Hb on the third postoperative day in the IELD group compared with the preoperative Hb was significant, **P < 0.01.

### Clinical outcomes

Postoperative VAS and ODI scores decreased significantly in the two groups compared with their preoperative scores (*P* < 0.01). No statistically significant differences in VAS and ODI scores at 3 days, 3 months, 6 months, and 12 months after surgery were noted upon comparing the two groups (*P *> 0.05) (Table 3).

**Table 3:** Comparison of clinical outcomes of UBE/BESS and IELD.

	UBE/BESS	IELD	Statistical values	*P*
VAS back				
Preoperative	3.95 ± 3.00	3.22 ± 2.88	Z = −1.204	>0.05
3 days after surgery	1.05 ± 0.85*	0.82 ± 0.75*	Z = −1.282	>0.05
3 months postoperatively	0.57 ± 0.77*	0.58 ± 0.67*	Z = −0.329	>0.05
6 months postoperatively	0.38 ± 0.54*	0.40 ± 0.61*	Z = −0.038	>0.05
12 months postoperatively	0.29 ± 0.46*	0.38 ± 0.49*	Z = −0.948	>0.05
VAS leg				
Preoperative	8.14 ± 1.26	7.82 ± 1.7	Z = −0.497	>0.05
3 days after surgery	0.90 ± 0.79*	1.04 ± 0.83*	Z = −0.740	>0.05
3 months postoperatively	0.79 ± 0.78*	0.94 ± 0.68*	Z = −1.104	>0.05
6 months postoperatively	0.74 ± 0.73*	0.78 ± 0.74*	Z = −0.280	>0.05
12 months postoperatively	0.43 ± 0.59*	0.38 ± 0.53*	Z = −0.297	>0.05
ODI				
Preoperative	66.07 ± 13.48	71.48 ± 15.94	t = −1.74	>0.05
3 months postoperatively	14.57 ± 6.66*	16.82 ± 6.17*	Z = −1.268	>0.05
6 months postoperatively	8.81 ± 5.84*	10.70 ± 6.21*	Z = −1.022	>0.05
12 months postoperatively	4.98 ± 3.11*	5.86 ± 3.73*	Z = −1.156	>0.05

Notes: (1) Within-group comparison of patients; the differences in VAS and ODI scores at each postoperative time point compared with preoperative scores were significant,*P < 0.01. (2) There was no significant difference in the VAS and ODI scores of waists and legs at each time point between the two groups of patients, *P* > 0.05.

**Figure 3:**
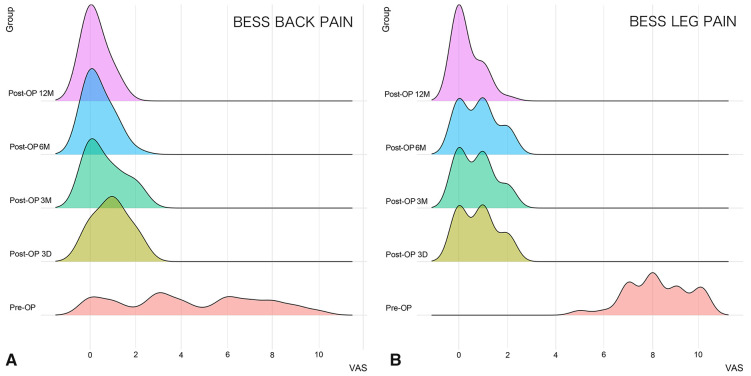
(**A**) Comparison of VAS scores for postoperative low back pain in the UBE/BESS group at each time point; no significant decrease was noted at 3 months postoperatively compared with that at 3 days postoperatively, and no significant decrease was noted at 12 months postoperatively compared with that at 6 months postoperatively (*P *> 0.05). Further significant decreases at 6 and 12 months postoperatively compared with that at 3 days and 3 months postoperatively were observed (*P* < 0.05). (**B**) On comparing the VAS scores for postoperative leg pain in the UBE/BESS group at each time point, no significant differences were noted at 3 days, 3 months, and 6 months postoperatively (*P *> 0.05), and a significant decrease at 12 months postoperatively compared with that at 3 days, 3 months, and 6 months postoperatively was observed (*P *< 0.05).

**Figure 4:**
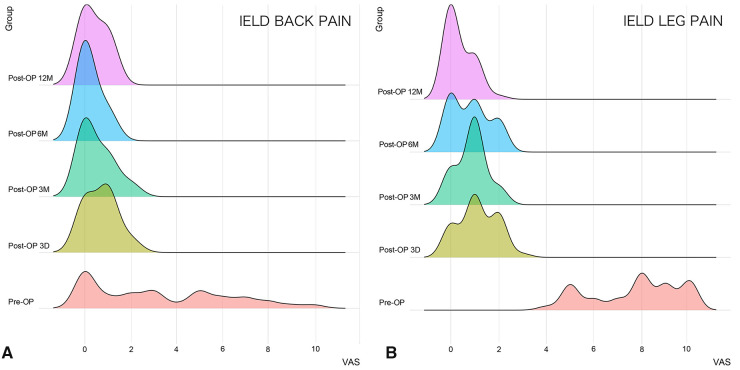
(**A**) Comparison of VAS scores for postoperative low back pain in the IELD group at each time point; no significant differences were noted at 3 days and 3 months postoperatively (*P* > 0.05). A significant decrease occurred at 6 months postoperatively compared with that at 3 days and 3 months postoperatively (*P *< 0.01). No significant difference was noted between 12 months postoperatively and 3 days, 3 months, and 6 months postoperatively (*P* > 0.05). (**B**) Comparison of VAS scores for postoperative leg pain in the IELD group at each time point; significant decreases in VAS scores for postoperative leg pain were noted at 12 months postoperatively compared with that at 3 days, 3 months, and 6 months postoperatively (*P *< 0.01). No difference was observed between 6 months postoperatively and 3 months postoperatively (*P *> 0.05); however, a significant decrease occurred in both groups compared with that at 3 days postoperatively (*P *< 0.05).

**Figure 5:**
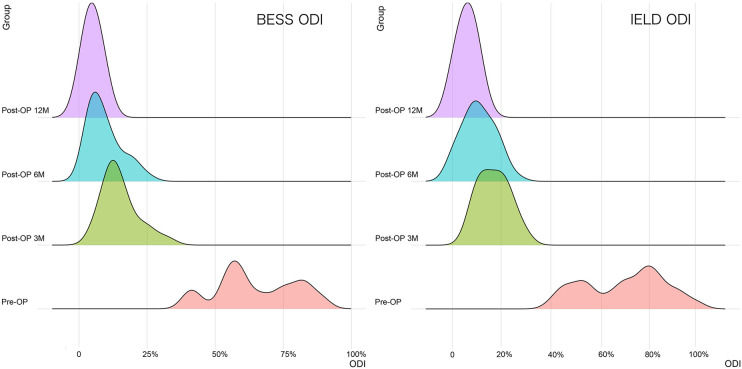
In the two groups, significant differences in postoperative ODI were noted at each time point (*P *< 0.01), and the ODI decreased significantly with time.

The distribution and comparison of the VAS scores for low back/leg pain in the UBE/BESS group at each postoperative time point are shown in Figure 3, and those in the IELD group at each postoperative time point are shown in Figure 4. The distribution and comparison of the ODI at each postoperative time point between the UBE/BESS and IELD groups is shown in Figure 5.

### Complications and recurrence

One case incurred a dural tear in the UBE/BESS group. The patient had no postoperative symptoms and did not complain of discomfort upon wearing a lumbar brace to enable mobility on the second day after surgery. Moreover, no special treatment was administered. In the IELD group, one case developed recurrence 7 months after surgery, with symptoms similar to those before surgery, and the diagnosis was confirmed by MRI. The patient recovered well after the revised endoscopic surgery.

## Discussion

Lumbar disc herniation is a common disease in spine surgery, and it predominantly affects the L5/S1 segment (22, 23). The L5/S1 interlaminar window is large and located at approximately the same level as the L5/S1 intervertebral space (24); therefore, posterior endoscopic spinal surgery through the interlaminar window is more advantageous for the treatment of L5/S1 disc herniation. Therefore, IELD has been used to treat L5/S1 lumbar disc herniation in several previous studies (12–17). IELD was initially proposed by Professor Rutten in 2006 (25). In the same year, Gun Choi (26) reported the treatment of L5/S1 disc herniation *via* the interlaminar approach, achieving favorable clinical results. Since then, IELD has rapidly developed and emerged as a reliable technique for minimally invasive spine surgery. Because the operating channel of uniportal endoscopy is integrated with the endoscope, the surgical instruments involved are more slender than traditional surgical instruments, and the surgical procedure differs significantly from traditional surgery. Studies have increasingly shown that uniportal endoscopy is more difficult to master (18, 19). The biportal endoscopy technique was initially proposed in 1996 by De Antoni (27). By 2013, Soliman (28) had introduced the pump irrigation system to biportal endoscopic spinal surgery and proposed “irrigation endoscopic discectomy.” In 2017, Heo (29), for the first time, named the unilateral access biportal spinal endoscopy technique “Unilateral Biportal Endoscopy.” However, some scholars also called it “Biportal Endoscopy Spine Surgery (BESS)” (30–32). At present, both UBE and BESS represent biportal endoscopic spinal surgery (33). Since then, this technique has been rapidly developed by spine surgeons worldwide through continuous research and improvement. Many studies have demonstrated favorable clinical results from UBE/BESS and IELD in the treatment of lumbar disc herniation; nevertheless, research on the possible differences between the two techniques remains limited.

UBE/BESS has unique features compared with IELD: (1) In UBE/BESS, conventional arthroscopes and surgical instruments can be utilized to complete the surgical procedure without purchasing a special uniportal endoscopic system or supporting surgical instruments. (2) UBE/BESS involves two channels. The endoscope and operating instruments are in different channels, which can move independently and freely. This significantly increases the observation range of the endoscope and working area of the surgical instruments. (3) The surgical path and decompression process of UBE/BESS are similar to those of conventional microscopic lumbar discectomy, and studies have demonstrated that the learning curve of the UBE/BESS technique for lumbar disc herniation is 14 cases (31). (4) The distal operating channel of UBE/BESS is not restricted by a rigid working cannula, thus allowing the use of conventional, large-sized surgical instruments, such as an osteotome, rongeur, nucleus pulposus forceps, and nerve retractor, among others, and greatly improving the working efficiency.

Due to the lack of a rigid cannula to dilate the soft tissue in the UBE/BESS technique, blunt dissection of the muscle is required to create a working space before decompression of the spinal canal. Therefore, theoretically, UBE/BESS should result in greater blood loss and worse postoperative back pain than IELD. Certain studies have attempted to address these issues. Hao (34) retrospectively analyzed 40 patients with simple L4/5 disc herniation treated with endoscopy between 2018 and 2021, including 20 cases of UBE/BESS and 20 cases of uniportal endoscopic spinal surgery. In terms of intraoperative blood loss, operative time, postoperative hospital stay, and postoperative pain, uniportal endoscopic spinal surgery was superior to UBE/BESS. Jiang (35) retrospectively analyzed 54 cases of single-segment lumbar disc herniation treated with spinal endoscopy, including four, 33, and 17 cases of the L3/4, L4/5, and L5/S1 segments, respectively. All patients were divided into two groups: 24 and 30 patients in the UBE/BESS and uniportal endoscopy groups, respectively. One dura tear occurred in the UBE/BESS group, and no statistically significant differences were noted in terms of clinical outcome, pain control, and patient satisfaction among the patients in both groups. In this study, the researchers calculated the total surgical blood loss of patients based on hematocrit change before and after surgery and found the total blood loss in the UBE/BESS group to be significantly greater than that in the uniportal endoscopy group. In addition, the UBE/BESS group had a larger surgical incision, longer operative time and hospital stay, and higher total medical costs.

To the best of our knowledge, these are the only two studies to have compared the clinical efficacy of UBE/BESS with that of uniportal endoscopy in the treatment of lumbar disc herniation. However, the above two studies had certain shortcomings in terms of trial design. For example, patients in the control group were operated *via* the lateral foramen, which differed from the surgical path of the UBE/BESS technique; the control group was operated on under local anesthesia, whereas the trial group was operated on under general anesthesia; the trial group was operated on in the prone position in one study, whereas the control group was operated on in the lateral position; and the surgical segments in one of the studies were not similar in both groups. In the present study, we limited the surgical segment to the L5/S1 segment, and patients in both groups were operated on in the prone position under general anesthesia; moreover, both groups used a posterior trans-interlaminar approach to increase homogeneity, reduce trial bias, and improve the accuracy of the study.

According to our data, both the UBE/BESS and IELD groups achieved favorable clinical results, and postoperative low back pain, leg pain, and ODI scores significantly improved. No significant differences were noted between the two groups, exhibiting consistency with the two studies mentioned above (34, 35). We also found no difference between the two groups in postoperative low back pain, thus conflicting with Hao's results (34) but exhibiting consistency with Jiang's findings (35). This may be related to the fact that we performed blunt stripping of the spinous process lamina migrans when creating the working space in UBE/BESS and used this gap for anatomical identification after placement of the endoscope to rapidly enter the interlaminar window with minimal damage to the multifidus muscle. No significant difference in intraoperative radiation exposure was noted between UBE/BESS and IELD. In terms of operative time, no statistical difference in the overall operative time was observed between the two groups; nonetheless, we categorized the overall operative time based on our decision to enter the spinal canal as a marker and recorded the extracanal operative and intracanal decompression times as well. We found the extracanal time in the UBE/BESS group to be significantly longer than that in the IELD group, while the intracanal decompression time was significantly shorter than that in the IELD group, a phenomenon that reflects the difference between the two techniques during implementation. UBE/BESS required more time to identify the tissue structure and enlarge the working space when operating outside the spinal canal; however, nucleus pulposus removal and nerve root release proved more efficient after entering the spinal canal due to the operating habits and equipment. While IELD required significantly less time to operate outside the spinal canal because of the role of the rigid cannula, the inefficiency of the instruments and difference in operating habits prolonged the removal of the nucleus pulposus and fibrous ring after entering the spinal canal.

In terms of surgical trauma, the difference in postoperative hospitalization time between the two was not significant. The surgical incision length in the UBE/BESS group was significantly longer than that in the IELD group, and the pre- and postoperative Hb change was significantly greater in the UBE/BESS group than in the IELD group, indicating that the actual blood loss in the UBE/BESS group was greater than that in the IELD group. Because both endoscopic surgical techniques require intraoperative saline irrigation, the intraoperative blood loss could not be accurately estimated. Furthermore, the postoperative “hidden” blood loss could not be estimated because no drainage tube was used after surgery. Therefore, in this study, we selected the method of dynamic Hb monitoring to evaluate the actual postoperative blood loss. Certain studies have shown that dynamic monitoring of hematocrit and Hb can effectively and accurately reflect blood loss in surgical patients. Both are potentially useful in calculating the actual blood loss after surgery (36, 37). To reduce the influence of iatrogenic causes, such as preoperative, intraoperative, and postoperative transfusion effects on Hb, strict fluid and medication management were performed on all patients to render the two groups as homogeneous as possible and improve the accuracy of the study. Considering the length of the surgical incision and postoperative Hb level changes, we concluded that the UBE/BESS technique was more invasive than the IELD technique; nevertheless, it did not significantly affect the postoperative recovery time of patients.

This study has certain limitations. First, it is a retrospective study with a short follow-up time and a small sample size. Second, only L5 and S1 segments were compared in this study. In addition, IELD was the exclusive control procedure in this study, whereas microscopic discectomy is also an effective, minimally invasive method for the treatment of lumbar disc herniation. Therefore, these minimally invasive surgical techniques should be discussed together in future studies.

In conclusion, the UBE/BESS and IELD techniques are both safe and effective in the treatment of lumbar disc herniation. The introduction of dynamic Hb in this study revealed that IELD involves less actual blood loss and trauma for the patient than UBE/BESS, suggesting that UBE/BESS requires optimization in the future to further reduce trauma. The rigid cannula used in IELD potentially reduces the extracanal operative time. The surgical equipment used in UBE/BESS is more efficient in removing the nucleus pulposus and releasing the nerve roots.

## Data Availability

The raw data supporting the conclusions of this article will be made available by the authors, without undue reservation.
